# Smart Co-housing for People With Disabilities: A Preliminary Assessment of Caregivers’ Interaction With the DOMHO System

**DOI:** 10.3389/fpsyg.2021.734180

**Published:** 2021-09-03

**Authors:** Davide Bacchin, Patrik Pluchino, Adriana Zoe Grippaldi, Daniela Mapelli, Anna Spagnolli, Andrea Zanella, Luciano Gamberini

**Affiliations:** ^1^Department of General Psychology, University of Padova, Padova, Italy; ^2^Human Inspired Technology Research Centre, University of Padova, Padova, Italy; ^3^Department of Information Engineering, University of Padova, Padova, Italy

**Keywords:** internet of things, co-housing, caregivers, disabilities, user experience, accessibility

## Abstract

Millions of people with motor and cognitive disabilities face hardships in daily life due to the limited accessibility and inclusiveness of living spaces which limit their autonomy and independence. The DOMHO project deals with these fundamental issues by leveraging an innovative solution: a smart co-housing apartment. Besides, the project aims at exploiting the well know effects of co-housing on individuals’ health and well-being in combination with ambient assisted living technologies. The present study focused on the interaction of caregivers with the control application of an integrated smart system. Participants performed different tasks, fill out a questionnaire, and were interviewed. Performance and usability of the user interface, trust in technology, privacy, and attitudes towards home automation were explored. A series of guidelines for domotic technology control interfaces design was identified, and a high level of trust in these advanced tools was shown. Caregivers considered smart technologies as a work aid and a means for enhancing autonomy and life quality for users with disabilities.

## Introduction

To date, the World Health Organization (WHO) estimates that at least 1 billion people in the world are affected by some form of disability, which corresponds to about 15% of the entire population above 15years old (World Health Organization, 2020). These numbers are still growing, given the increased population’s life expectancy and related health diseases. Insofar as disabilities are a relevant issue in our society, significant efforts must be made to support the health and well-being of individuals that are affected by these conditions. WHO reports the need to help the elderly and people with disabilities, addressing the necessity to overcome healthcare costs, limited access to resources and services, and physical barriers. Moreover, older people and individuals with disabilities are more prone to face risks connected with loneliness and social isolation, which might have catastrophic effects such as increased mortality, susceptibility to dementia, poor self-rated physical health (Dickens et al., 2011; Emerson et al., 2020). Therefore, efforts have been directed towards the research on innovative technological solutions to mitigate or solve the above-mentioned issues to increase the quality of life (QoL) of these individuals. In this regard, Smart Homes (SH) play a crucial role because they consider comfort, healthcare, safety, security, and energy consumption (Alam et al., 2012). These intelligent tools are conceived to allow greater accessibility and usability for a broad category of people, overcoming problems like limited access to services and physical barriers. Besides, these technologies can be integrated into networks to communicate with each other, adopting the Internet of Things (IoT) paradigm (Wan et al., 2017). IoT systems, for instance, allow the possibility of exploiting technological devices to monitor the variables linked to individuals, living spaces, and other technologies functioning to prevent or detect potential issues and supporting those who live in the environment when they need it (Jiang et al., 2004; Cena et al., 2019). The IoT feature also plays a fundamental role in the cost management of people with disabilities and the elderly, both in accelerating eventual medical interventions, for example, in case of seizures, and reducing the need for home assistance, thanks to reliable and constant monitoring. Moreover, the IoT paradigm could make possible a scaling-up economy to open the market to different players (Zanella et al., 2020). Nowadays, the market competition has permitted the development of devices such as the Google Nest, Amazon Alexa, and Apple HomeKit, which are increasingly used worldwide.

The present work describes a real-world trial comprised in the DOMHO project, which evaluated the interaction of professional caregivers with an advanced domotic system. The project’s overall objective was designing and developing IoT systems for ambient assisted living (AAL). The technological solutions are exploited to support several people in a domestic environment that adopt a particular model of sharing living spaces: the co-housing. Indeed, the project technologies permit the supervision of inhabitants by professional caregivers to prevents hospitalization, while the co-housing experience wants to mitigate issues related to loneliness and social isolation. The combination of co-housing and smart tools lays the basis for a supportive environment that could increases social protection, autonomy, and well-being for individuals with special needs and their caregivers.

### Co-housing to Avoid Social Distancing and Loneliness

The co-housing experiences in Europe and worldwide positively correlate with social inclusion and increasing feelings of well-being, self-efficacy, and esteem (Lubik and Kosatsky, 2019). It is currently impossible to exclude from this discussion the COVID-19 pandemic, which has inevitably worsened the health risks for people with disabilities. The 40% of adults with a disability or a chronic disease reported feeling lonely or socially isolated (CHRT, 2021). Significant risk factors for those conditions include living alone, motor disabilities, major life transitions, and emerging health problems. Besides, seniors reporting feeling lonely or social isolation have a 45% greater risk of mortality because these problems can negatively affect physical and mental well-being (Banerjee and Rai, 2020). For those reasons, it is necessary to evaluate the co-living experience to face loneliness and isolation and exploit its potential to significantly improve physical and mental health (Burgess and Quinio, 2018).

In co-housing history, a critical phase is the 1970’s movements that permit exploring new ways of living, sharing spaces with people’s social support. For many years co-housing had been seen as a “utopian dream,” too distant from reality. However, in the last decade, people have begun to consider this model of coexistence with renewed interest. Co-housing introduces the relevant concept of autonomy that does not exclude sharing. Vestbro and colleagues (Vestbro and Horelli, 2012) defined this experience of living together as: “housing of common space and shared facilities.”

The majority of the studies on co-housing involved older adults. One of the most extensive research (Jakobsen and Larsen, 2019) analyzed 110 co-housing communities in Denmark with two internet-based surveys that explore their daily life and the motivation of choosing such a lifestyle. Results showed that co-housing experience correlates with high life satisfaction. However, the authors stated that a considerable limitation of their study was that participants were all rich and privileged people. Another example is the United States co-housing community, analyzed by (Jenkins, 2017) in his research. He evaluates a series of co-housing communities’ websites and visits three communities to outline the crucial values of people that choose this sharing experience. The results show that caring (i.e., depth of relationships), community, diversity, and sustainability are considered fundamental dimensions to consider in designing supporting technology for co-housing.

In the Netherlands, Rusinovic and colleagues (Rusinovic et al., 2019) conducted a qualitative analysis in eight communities for the elderly, finding that co-housing leads to a reduced presence of social loneliness and an improved perception of the sense of affiliation and social and personal safety. Besides, (Brenton, 2013) highlighted the advantages of senior co-housing linked to active participation in a group of people. Indeed, it encourages the acquisition of a social role and compensates for the anonymity of the classical single households in which many older people live. Moreover, it appears that co-housing could be an additional option for informal care, reducing demand (and costs) for health and social services.

Considering people with disabilities or impairments, the ENEA Project (Maestosi et al., 2018) is an example of an SH based on the co-housing concept to create a replicable Smart Home Network model (SHN). SHN permits reducing energy consumption while providing traditional and innovative services for inhabitants. This project aimed at creating a secure environment in which people share space and are, at the same time, supported by the SH services and monitored remotely by qualified staff. Three essential services assure the improvement of life quality in helping people with disabilities: security, safety, and feature for increasing QoL.

In conclusion, the co-housing experience in a smart home environment could turn out to be a new living form to promote and support older people and individuals with disabilities to increase their autonomy and independence, to receive social support, and to feel safer.

### Smart Homes

The ever-growing interest in SH is justified by several advantages, starting from the reduction of energy consumption to the expected increase in well-being, QoL, social sustainability, home comfort, protection, and security (Marikyan et al., 2019; Schill et al., 2019). Furthermore, SH technology is considered a way to reduce care costs (Amiribesheli et al., 2015). In Europe, an analysis on the market of smart homes estimated a demand equal to 22.5 million in 2017, corresponding to 9.9% of European households (Berg Insight, 2021). The growth is attested in 30%/year and a forecast of 84 million at the end of 2022. A recent review (Sovacool and Furszyfer Del Rio, 2020) has identified 276 commercialized technologies on the market. They are classified into 13 categories: household appliances, lighting, energy and utilities, entertainment, health and wellness, safety and security, baby and pet monitors, clothes and accessories, vehicles and drones, home robots, gardening, integrated solutions, and “others.” Indeed, recent studies on SH aimed to assess the technological and economic factors, regardless of the potential social benefits of SH in improving people’s QoL (Marikyan et al., 2019). However, some studies focused on the users and on how automation can support people with disabilities to accomplish tasks and routines of daily living (Gentry, 2009). The automation of the house and the reduction of human involvement in these daily tasks increase the overall accessibility of the environment (Delnevo et al., 2018). In the next section, the exploitation of smart homes considering individuals with special needs is provided.

### Smart Homes for Individuals With Disabilities

According to a recent statistical analysis, the percentage of people in Italy with disabilities is 5.2% (Istat, 2019). These data suggest the enormous impact of disability and older age in our society, allowing us to understand the potential benefits of IoT technologies for AAL (e.g., people with severe motor disabilities that can remotely control doors, shutters, lights).

According to a recent review on intelligent technologies for AAL, smart homes should be adaptable, interactive, and contextual (Maskeliūnas et al., 2019). Technologies should recognize the context in which they operate throughout data and sensors to adapt their responses without direct user intervention. The system should also interact with individuals to better learn how to act correctly. Maskeliunas and colleagues also underlined that the different sensors, which describe the environmental state, could collect information on time, temperature, noise, pollution, and human data (e.g., human body language, requests, and needs). The intelligent system may exploit these data to assist humans and enhance their health, QoL, and comfort, thus potentially increasing technology acceptance. For example, older adults have a series of problems that AAL technologies can face, e.g., risk of fall, social divide, reduced well-being and independence (Moreno et al., 2014; Yusif et al., 2016). Moreover, as suggested by (Domingo, 2012): “We firmly believe that the IoT can offer people with disabilities the assistance and support they need, to achieve a good QoL […] Assistive IoT technologies are powerful tools to increase independence and improve participation.”

This new vision of the home automation system as a support for social and individual independence has led to the emergence of different studies that explore the relationship between SH and people with special needs. These last can be both elderly and people with disabilities because they often shared similar issues.

Regarding the elderly, a recent literature review (Marikyan et al., 2019) reported that SH could improve socialization and even help users to overcome the sense of isolation. Another systematic review (Pal et al., 2017) focused on the actual efficiency of SH as a tool to improve the QoL. In the context of health monitoring, it results in an enhanced feeling of safety, less fear, and anxiety. For instance, it serves older people to remember daily tasks (e.g., drugs assumption) and strengthen their independence. Other positive consequences of SH use for the elderly concerning the decrease of loneliness, the improvement of satisfaction, and well-being. Furthermore, using ICT technologies and caregivers’ help encourages self-independence (Pal et al., 2017). In a paper by Carnemolla (Carnemolla Bruno et al., 2018), SH technologies are also discussed in the context of interventions that would bring benefits in facilitating self-care and autonomy, supporting older people’s safety in the home by automating tasks with a reduction of the related risks.

Regarding people with disabilities, several examples of SHs implementation can be founded. The first example is the ENEA project already cited (Maestosi et al., 2018). In this project, a SH firstly provides services for user’s security, helping to detect when someone tries to break-in the locking system, providing alert notifications. Concerning safety, the SH network can monitor specific environmental parameters (smoke detectors, C02, flood sensors) to detect risky situations and prevent injuries and accidents. Finally, some assisted living features supporting people with a vulnerability to live a longer and better life in their own homes. This housing model is an approach that permits an adaptation of the smart home to the individual’s specific needs. In Japan, the Robotic Smart Home was designed and developed to increase the comfort, safety, and security of disabled and older people, using three robotic assistive systems (Tanabe et al., 2019). The first was a mobility and transfer assist system, helping people move freely around the house. The second system was an operational assistance system helping the inhabitants manage the house (e.g., opening curtains, turn on the TV, etc.) through a connection node with the other system that exploits IoT technologies. The third was an information assist system representing the connection with remote systems such as medical institutions or users’ physiological monitoring devices. Chen and colleagues (Chen et al., 2017) developed a control interface based on Morse code that allows controlling different smart devices. It was implemented, tested, and then evaluated for several months involving people with severe disabilities, up to total paralysis, obtaining favorable results considering system feasibility and interaction efficacy. Another project is the DAT (Andrich et al., 2006) which proposed an intelligent home environment for users with disabilities. DAT developed and evaluated clinical protocols and innovative system control solutions in an apartment consisted of seven rooms. The integrated technologies were finalized to promote independence, safety, and health monitoring of the people with disabilities and reduce caregivers’ burden.

The ProACT project (Malavasi et al., 2019), conducted in Italy, Ireland, and Belgium, proposed an ICT-based solution for people with special needs. Different intelligent tools were considered, e.g., air quality and physiological sensors (i.e., pulse oximeters and glucometers) and smart cameras. Also, in 2018, Enshaeifar and colleagues (Enshaeifar et al., 2018) described the Technology Integrated Health Management project, which integrates IoT devices into a single platform capable of communicating with caregivers. Thanks to wearable technologies, medical devices, and others, data are collected to inform operators about dementia patients’ clinical conditions. The study adopted a co-design approach to evaluate patients, caregivers, clinicians, and industrial partners. The system seems capable of taking care of patients thanks to its predictive systems. The possible detected problems could be urinary infections derived from bathroom use and temperature data or highlighting a dangerous event with the fall detection system.

Recent research proposed a framework that could allow people with different disabilities, such as blindness or deafness, to interact with the home environment. (Mtshali and Khubisa, 2019) detailed a system that utilized commercial voice assistants such as Amazon Alexa, Google Home, or Apple Siri to capture users’ voice commands to control the lighting system. Another study (Pradhan et al., 2018) supported the hypothesis of adopting commercial devices such as the Amazon Echo to help people with different disabilities in interacting with smart objects. For example, a study of Balasuriya and colleagues (Balasuriya et al., 2018) with 18 participants with special needs reported that, in 72% of cases, the condition of activating some commands utilizing voice-based interfaces was preferred over graphical interfaces. These results were confirmed by another study reporting that 16 people with disabilities could effectively operate a voice assistant also if they present a mild cognitive impairment, but they are capable of repeating simple sentences (Masina et al., 2020).

Besides, the benefits of SH also affect caregivers’, particularly minimizing adverse effects on their work-related stress (Machiko et al., 2010) and reducing their burden (Lindeman et al., 2020). For example, imagine a user with a motor disability becoming more autonomous and independent. As a result, the QoL of the family and the working condition of caregivers might improve. Indeed, different recent papers report the positive effect of assistive environments in reducing the perceived burden derived from the constant commitment and effort to care for individuals with disabilities (Dupuy et al., 2017).

### The DOMHO System

**Figure 1:**
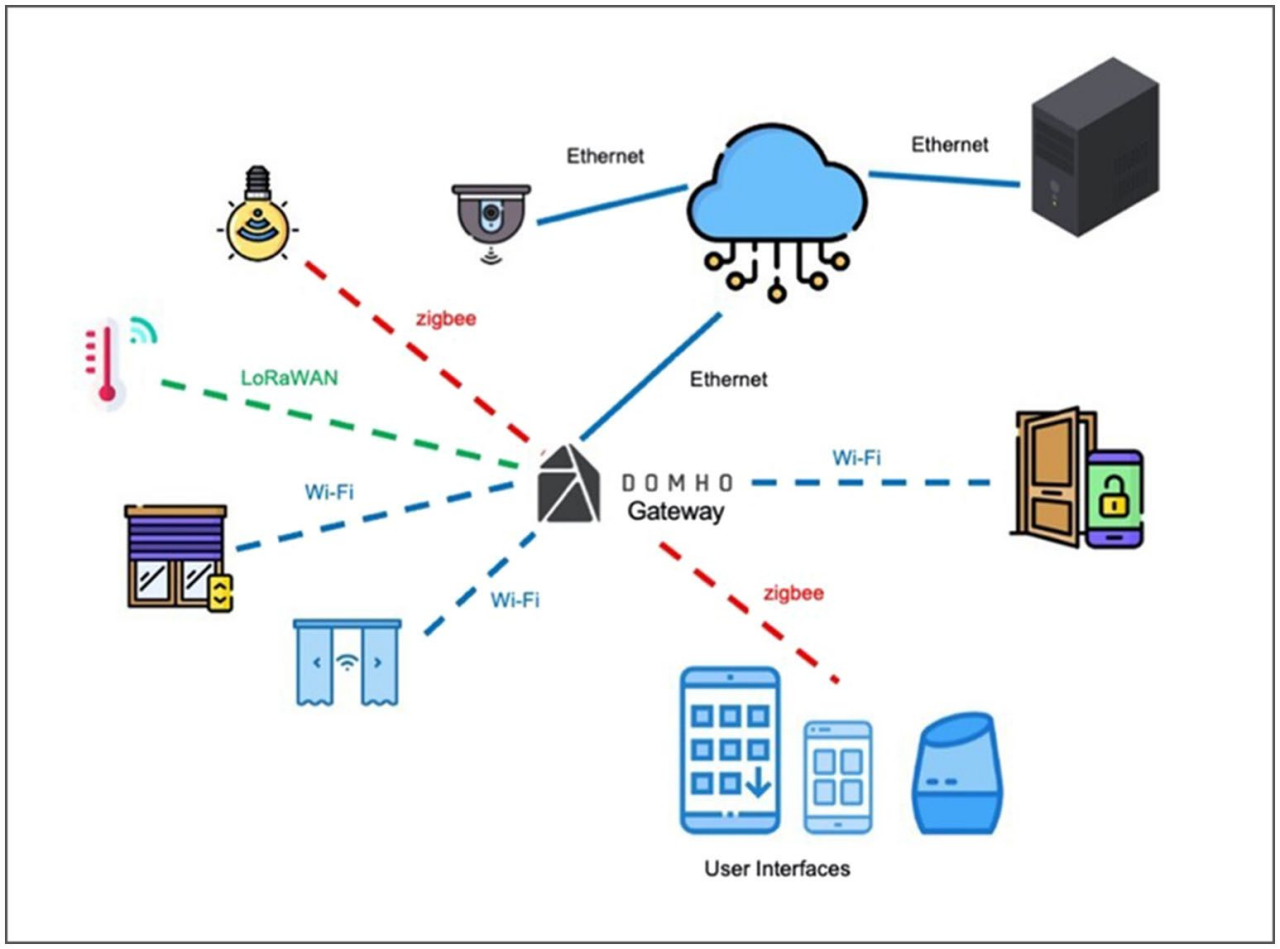
DOMHO system architecture.

A brief overview of the DOMHO project is provided. Zanella and collaborators (Zanella et al., 2020) presented a detailed description of the design and development of this IoT system. In synthesis, the system technologies can connect each other thanks to a smart gateway that permits exchanging sensor data and integrating them with the cloud information (Figure 1). Thanks to this gateway, the system’s different types of technologies can communicate despite exploiting different protocol languages and connections. This feature allows the technological solution to be flexible, safe, modular, and customizable. The system can control various types of devices, which have been divided into three categories: lightings, automations, and environmental sensors.

Similarly to other research works (Troussas et al., 2019; Wallisch et al., 2019) DOMHO adopted a user-centered approach. Crucial was the involvement of people with disabilities, older adults, and their caregivers in co-design sessions. The purpose was to gather their needs, expectations, desires concerning the features of assistive technologies (i.e., Focus Groups) and evaluate the control user interfaces (Zanella et al., 2020). Several intelligent technologies were considered to help people with disabilities and elderly. The project carried out trials in a smart co-housing apartment (i.e., real-world scenario) involving people with disabilities and their caregivers. The integrated IoT system allows controlling lights and automation in the apartment’s room, thanks to a user interface (i.e., smartphone, tablet application). Concerning the lighting devices, it is possible controlling their state (i.e., on/off), brightness intensity, colour and the temperature of the light (i.e., cold or warm). As for the automation (i.e., door, curtains, and shutters), it is possible to open/close them and stop their movement at every moment (except for the doors for safety reasons). In addition to the direct control of smart devices, the application allows to create usage scenarios (i.e., registered commands for multiple technologies to activate in specific usage situations) and customize the system operating to reduce the time needed to send commands to several technologies independently. These scenarios can be activated in manual mode (i.e., immediate start) or can be scheduled to begin automatically at a specific time (e.g., closure of all the shutters and curtains and turning off all the lights at 10p.m. each evening of the week). Finally, DOMHO comprehends machine learning algorithms embedded in a series of intelligent video cameras aimed to predict and prevent inhabitants from falling. They analyze the number, body positions and movements of the people in the environments. These data influence the functioning of lights and doors according to the people’s behaviour in the monitored environment.

In the following sections, a preliminary in-field trial involving seven professional caregivers is outlined. No elderly or individuals with disabilities were considered at this stage. The motivation that led to the completion of this study is twofold. Firstly, testing how the system was perceived by caregivers, focusing on aspects of perceived security, usability, ease of learning, and privacy protection, delegating job responsibilities to home automation, as essential factors for the acceptance of technologies (Gücin and Berk, 2015; Lah et al., 2020). Secondly, identifying a series of guidelines based on caregivers’ opinions and suggestions to inform designers and developers to build cutting-edge systems to improve the quality of operators’ work and of the life of people they care for.

The present study’s objective is to evaluate in terms of performance, user experience, intention of usage, learnability, and risks perception, the interaction of caregivers with a technologically smart apartment for co-housing.

The research questions of this study are:

- RQ1 - Is the interaction of caregivers with the application linked to positive perceptions of user experience, usability, privacy, security, and trust?
- RQ2 - Is the general perception of a domotic system positive, considering benefits, ease of use, risks, and the possibility to assign to the system some responsibilities?

Indeed, the hypothesis are:

- H1: We predicted that the evaluation of the control application would be related to an overall positive user experience, with a high level of usability, privacy, security, and trust. Insofar as the design and development of the application involved the participants directly, considering their needs and desires, directly following the principles of usability.
- H2 - We expect a positive overall attitude towards the system, characterized by low level of risks perception and a high trust. Being involved in the participatory design activities and knowing the IoT system’s potentiality will be a relevant factor.

## Materials and Methods

### Participants

Seven professional caregivers (*F*=7, M_age_=31; SD_age_=13) took part in the experiment on a voluntary basis. These individuals work in a daycare centre for people with disabilities. The mean work experience in the educational field is 13years (SD=12years). Overall, the sample had experience in smartphone use (*M*=9.8years; SD=2.5years), the majority (*N*=5) use voice commands at least once a week, and one participant has experience with commercial home automation (e.g., Amazon Echo).

### Materials and Methods

The present study exploited a mixed approach to assess user experience and usability of the intelligent domotic system and its control interface. The following quantitative and qualitative tools were considered:

- Computer-supported video analysis (i.e., BORIS software; Friard and Gamba, 2016) to evaluate the performances and the overall interaction of participants with the application.
- An *ad hoc* User Experience (UX) questionnaire to assess participants’ user experience and usability. The instrument (Supplementary Material presents the items) took into account the following dimensions: Pleasantness, Privacy, Recognition Rather Than Recall, Satisfaction, Security, Trust, Usability, and Visibility of the System Status. It consisted of 23 items on a 5-point Likert scale.
- A semi-structured interview with four open-ended questions.

Several instruments were utilized in the experiment. The application that allows the control of all the smart devices was installed on a Samsung S8 Smartphone (screen 5,8″, resolution: 1440×2,960 pixel). A GoPro Series 4 camera (GoPro^®^) and a flexible tripod (GorillaPod; Joby^®^) were utilized to video-record the experimental sessions and permit the offline computer-supported video analysis. Finally, a Shure MV88 digital iOS condenser microphone was used, paired with an Apple^®^ iPhone^®^ 12 mini, to record (application MOTIV Audio, Shure^©^) the interviews.

### Experimental Tasks

Two apartment areas were used for carrying out the experiment: the living room (i.e., an open space that also comprises the kitchen) and two communicating bedrooms. Participants were asked to accomplish four different tasks utilizing the provided smartphone for interacting with the IoT devices of the smart apartment. Two tasks were performed in the living room while the others two in the bedrooms. In the first task (i.e., T1, living room), participants should control single devices in the manual mode through the application (i.e., immediate effect). They had to control the lights (i.e., switching on and intensity) and the automation (i.e., curtains and shutters). The second task (T2, living room) required first to create a manually activated scenario (i.e., turn on all the lights and close all the automation), add it to the preferred scenarios menu, and activate it. The third task (T3, bedrooms) involved the closure of one door and the manual modification of lights through the application (i.e., state: on/off, intensity: 0–100, color: green/white/red, temperature: cold-warm). In the last task (T4, bedrooms), participants had to create an automated scenario (i.e., all lights turned off and all automation opened at 8.00 every day of the week). The order of the rooms’ and tasks’ presentation was counterbalanced across participants.

### Procedure

The week preceding the experiment, the participants carried out a short training that presented the entire IoT system and its general functioning that lasted around 45min. During this training, they were also able to explore and test the system freely.

**Figure 2:**
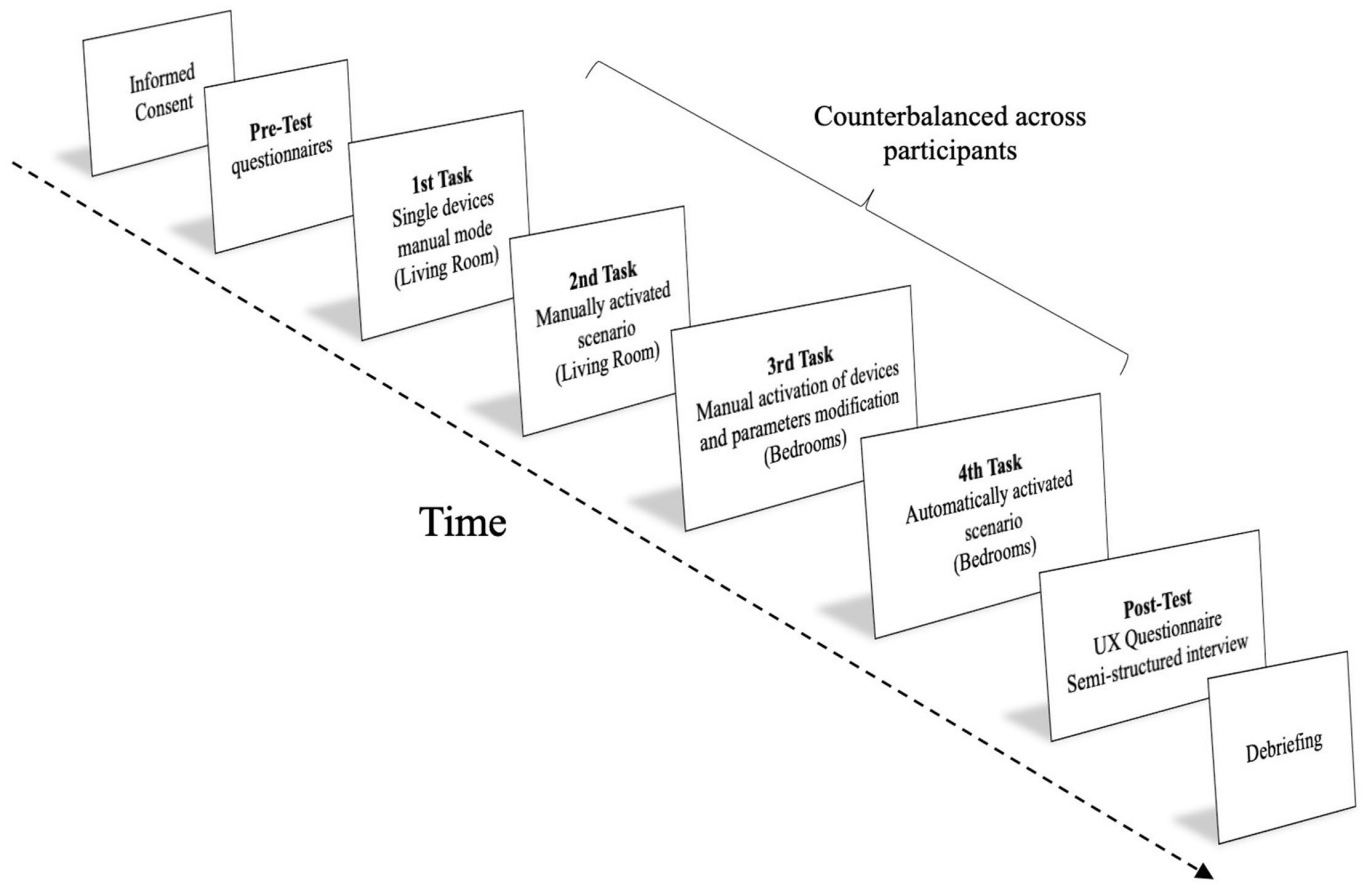
Graphical depiction of the experimental procedure.

After a week, the caregivers arrived at the apartment to perform the preliminary trial. Each participant had to fill out an informed consent and a short demographic questionnaire formulated to gather background information (i.e., age, gender, and frequency of smartphone use). A five-minute free exploration of the system allowed the caregiver to familiarize again with the application before the trial. Then, each participant performed all the tasks while the interaction with the smartphone was video recorded using a GoPro Series 4 camera (GoPro^®^) and a flexible tripod (GorillaPod; Joby^®^). Then, each participant filled out the *ad hoc* UX questionnaire. Finally, the four open-ended questions of the semi-structured interview (i.e., audio-recorded) were administered. The questions concerned caregivers’ attitudes, intention, and motivation of using smart technologies to support people with disabilities and improve their working-life quality. The whole experimental session, summarized in Figure 2, lasted approximately 35min.

## Results

Utilizing the age variable collected using the demographic questionnaire, it was possible to split the sample into two groups, respectively, above (*N*=3) and below (*N*=4) the median (Mdn=26) of the age. Overall, in the case of series of tests the *p*-values were adjusted with the Benjamini-Hochberg method (BH; Benjamini and Hochberg, 1995).

### Video Analysis

The video recordings of participants’ interaction with the control application permitted them to evaluate the various actions accomplished to complete each of the four tasks.

Participants’ behaviors were analyzed in terms of number of the taps errors for each task, breakdowns occurrences (i.e., any critical moment in which the interaction slowed down or stopped; Gamberini et al., 2013), and time on task. Moreover, a descriptive analysis of the average percentage of task success was conducted. The data of the performance in terms of the number of physical interactions (i.e., taps) is shown in Table 1.

**Table 1:** Tasks performance: required minimum N^°^ taps, taps errors, percentual tap errors.

	Required taps	Error taps P01	Error taps P02	Error taps P03	Error taps P04	Error taps P05	Error taps P06	Error taps P07	% Errors/taps
Task 1	17	4	5	11	3	10	6	21	32
Task 2	37	21	36	1	5	25	4	3	29.5
Task 3	32	21	54	11	5	4	10	2	28
Task 4	52	49	28	12	13	0	9	6	23

**Figure 3:**
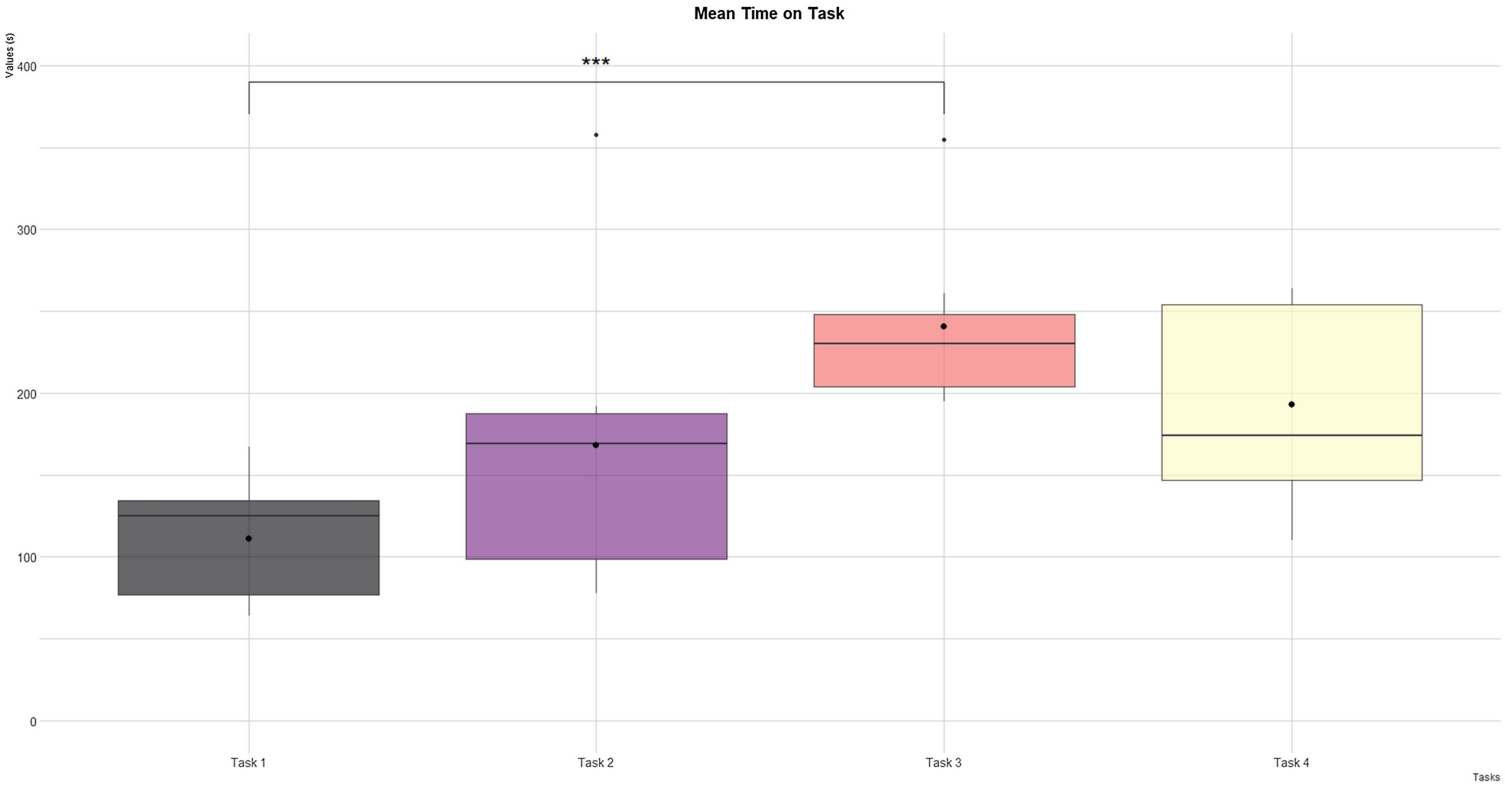
Mean time on task obtained in each proposed task. ^***^ = *p* < 0.001.

The analysis outcomes on time on task (i.e., the time required to accomplish each task) are shown in Table 2 and depicted in Figure 3. A series of t-test was conducted. The only significant difference was founded between T1 and T3 (*t*=−5.04, *p*<0.01). T1 and T3 were similar and easier tasks (i.e., controlling single devices); however in T3 the time on task was longer. Besides, a t-test has been conducted to evaluate the impact of age. However, a difference did not emerge (*p*>0.05).

**Table 2:** Task performance: time on task for each participant, mean time on task, standard deviation, median time on task.

	Time P01 (s)	Time P02 (s)	Time P03 (s)	Time P04 (s)	Time P05 (s)	Time P06 (s)	Time P07 (s)	Mean time (s)	SD	Median time (s)
Task 1	70	84	125	64	133	136	167	111	39	125
Task 2	169	358	93	192	183	78	104	168	96	169
Task 3	195	355	261	213	235	230	195	241	56	230
Task 4	250	171	110	174	123	258	264	193	65	174

**Figure 4:**
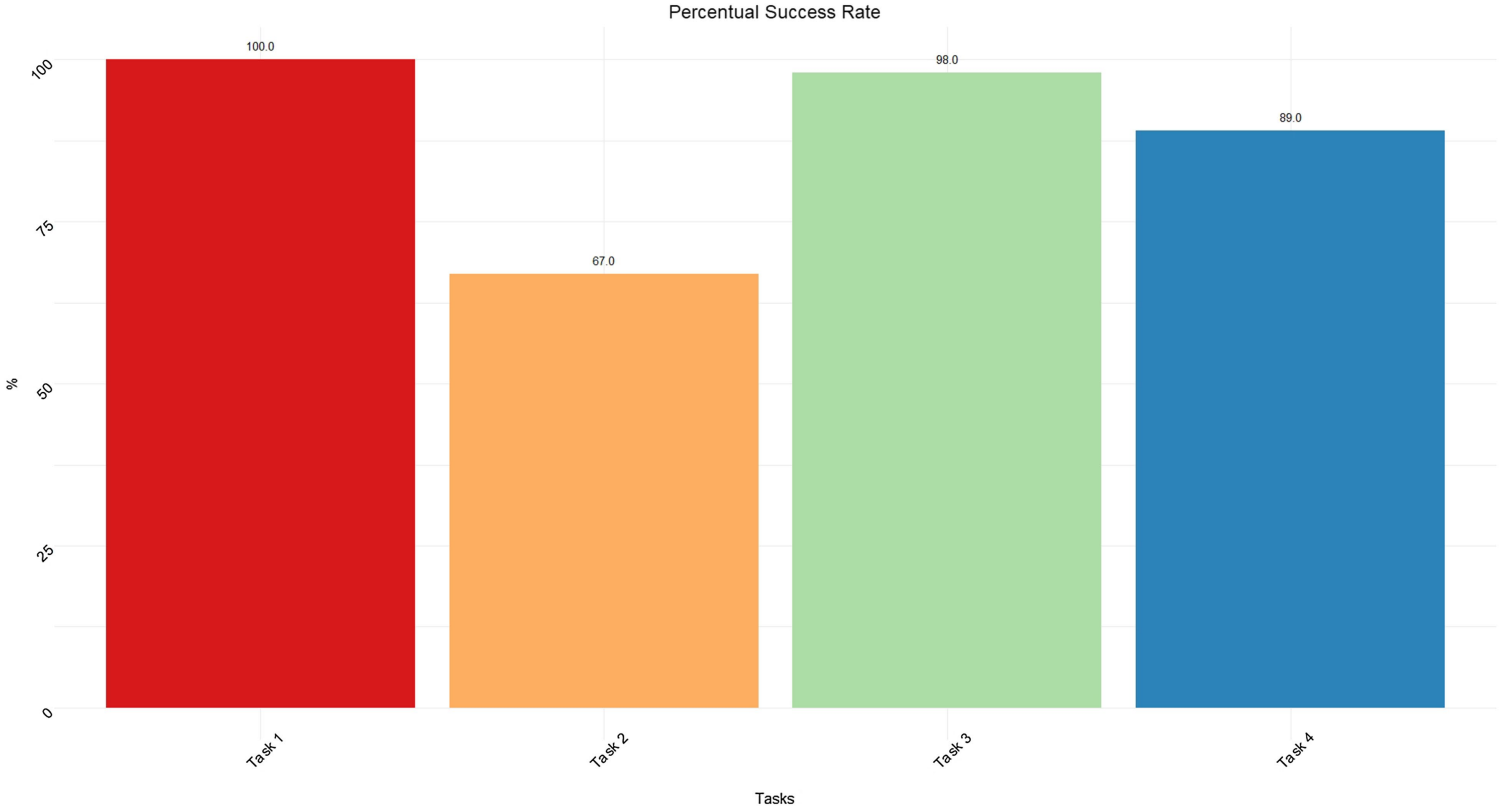
Percentual success rate for each task.

Moreover, Figure 4 shows the percentage of success in completing the experimental tasks. T1 and T3 (i.e., success percentage >98%) were accomplished almost perfectly, while T4 and especially T2 seem to present a lower level of success (respectively 89 and 67%).

**Figure 5:**
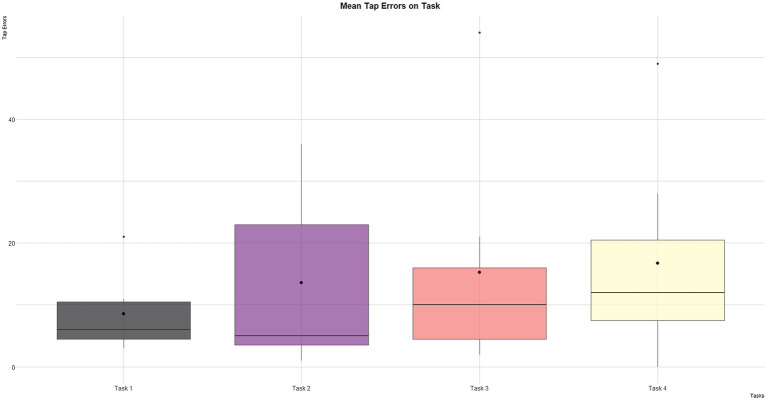
Mean number of errors for each task.

**Figure 6:**
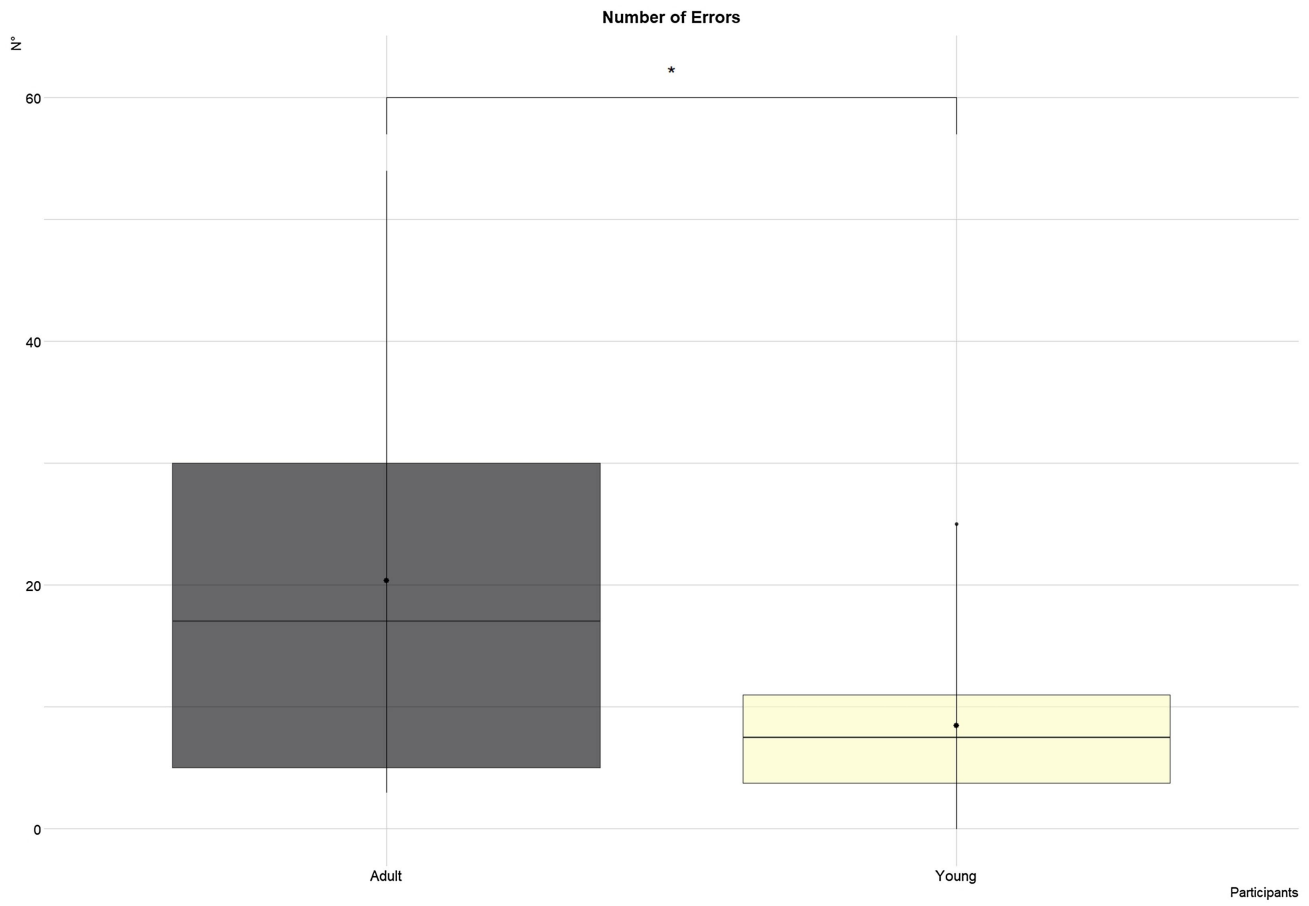
Number of errors as a function of age. ^*^ = *p* < 0.05.

Considering the number of tap errors, no significant differences were founded across tasks (Figure 5). Overall, a similar amount of mistakes were made (i.e., <17). The average errors committed by young (*M*=8.4, SD=6.9) and adult participants (*M*=20.3, SD=18.1) are shown in Figure 6. A trend towards significance emerged (*t*=2.2, *p*=0.05).

Regarding the breakdowns, two participants showed interaction difficulties in T2 linked to a misunderstanding of the feedback of the light state (on/off). One of these participants experienced a breakdown in T3 due to a doubt relate to labels of the bed lights. A third participant had a breakdown that lasted 90s attempting to create a scenario in T2 by the home page.

### UX Questionnaire

**Figure 7:**
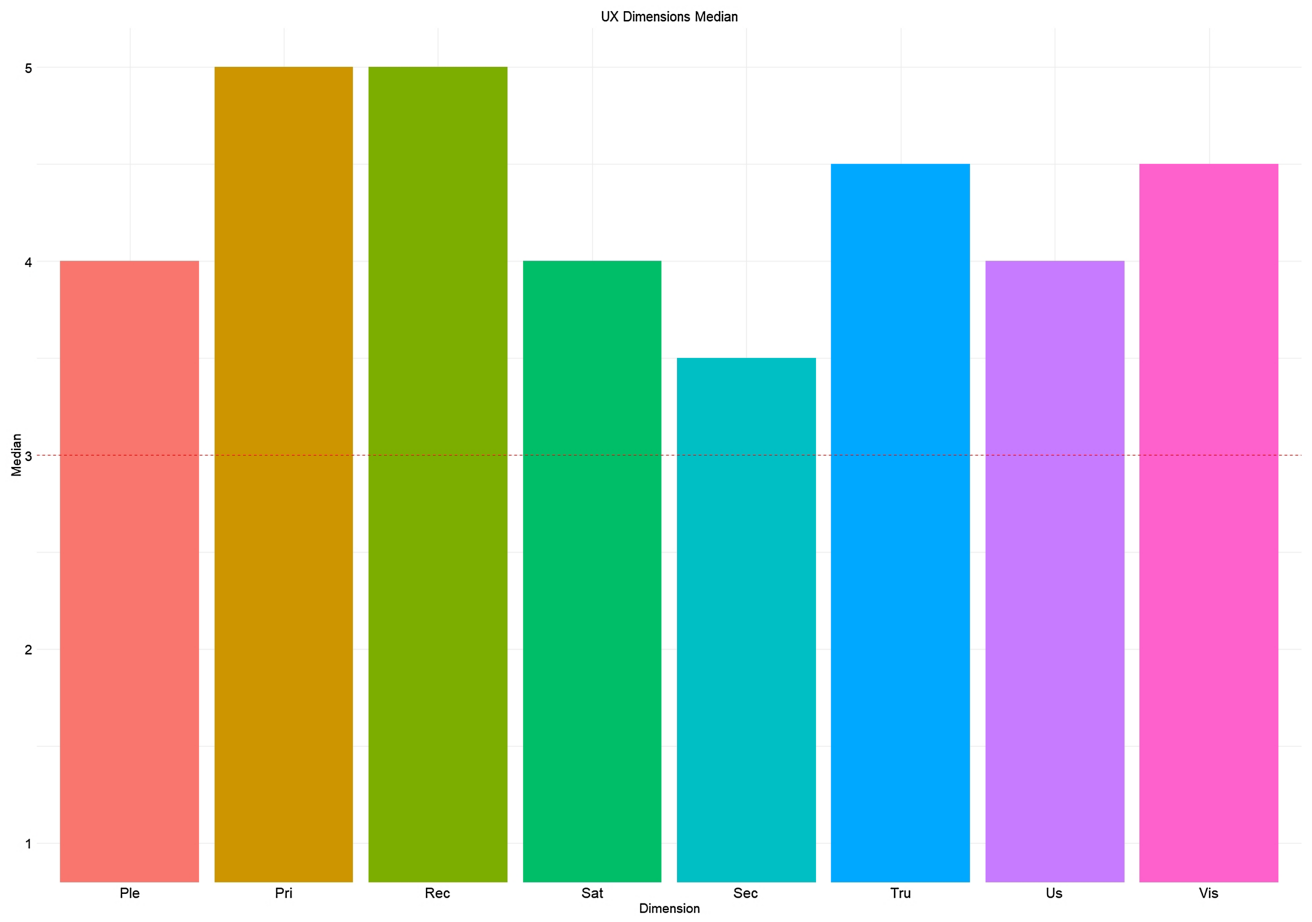
UX questionnaire. The labels for the dimensions are: Ple = Pleasantness, Pri = Privacy, Rec = Recognition Rather Than Recall, Sat = Satisfaction, Sec = Security, Tru = Trust, Us = Usability and Vis = Visibility of the System Status.

The participants evaluated the interface by assigning scores very close to the scale maximum for all dimensions (see Figure 7). The median of each questionnaire dimension was tested using one-sample Wilcoxon tests against the median value of the scale (Mdn=3). No differences emerged (all p>0.05). Finally, the analysis performed with a series of Mann–Whitney tests, considering the effect of age on the UX dimensions, did not show differences.

### Interview

The semi-structured interview included four questions that investigated the reasons for future use, potential risks, ease of use of smart technologies, and a particular aspect of acceptance, namely the operator’s responsibility. The interviews were transcribed and analyzed using the thematic analysis with a deductive approach, dividing the respondents’ answers according to the emerged topics and analyzing their frequency and content (Braun and Clarke, 2006). Each question and the relative analysis are detailed in the following sub-sections.

*Q1 - After using the application, do you think that you would be intended to use these tools in your work? What are the reasons that would promote you to use them?*

An overall agreement was related to the intention of using these technologies in their work. A greater enthusiasm appears in the statements of young operators (P03: “*Oh yes, yes yes yes”*; P06: *of course yes, it would be a significant help, to the users and also for the caregivers*) compared to the older ones (P01: “*So, yes, after trying I would like to use the same things*”; P02: “*on the part of the operators I think so*”), which, however, show feelings of caution but optimism. This can also be found in the words of P02, which defines the age and her low habits to technologies as fundamental factors for the acceptance of technology (P02: “*the limits for us operators, chronological age and history in the use of these means can make the operator a little more reluctant”*). To what concerns the reasons that would promote the use of IoT systems, it is interesting to note that operators initially answered from the perspective of people with disabilities and not for themselves. Participants stressed the importance of the system for promoting the autonomy of the individuals with disabilities (P01: “*to promote the autonomy of people*”; P02: “*they make the person more independent and more autonomous*”; P04: “*see and enjoy with the people what they can do independently*”; P05: “*because the technologies can give the autonomy that they need”*). Instead, from their point of view, the caregivers would use the system to support the working or daily activities (P01: “*it facilitates my professionalism*”; P06: *“technologies can reduce useless activities”*; P07: *“They make many things easier for you if by simply pressing a button all the lights are turned on, or the shutters lowered”*) and the reduction of workloads and anxiety (P04: “*I have a lower load, it lightens the anxiety and heaviness factor of the work*”). Furthermore, the safety systems have been identified as capable of providing help for greater attention to the people with disabilities and preventing accidents (P03: “*it can help me, as for a fall … to have greater attention and prevent a dangerous situation*”).

*Q2 - What do you think about the potential risks linked to these technologies?*

The operators highlighted how the general concern is linked to the potential incorrect functioning of the smart technologies (P01: “*an uncontrolled activation of scenarios or some aspects*”; P04: “*Non-functioning is a risk*”; P07: “*at the end, it is not a risky situation. Maybe only the non-functioning of the system could be a risk*”). The remaining operators were worried about system failures due to infrastructures, such as the supply of electricity and the internet, on which the system depends and which have their intrinsic reliability. If these systems fail, the participants were worried that this would not allow the system to work (P02: “*if you do not have the current and you cannot open”*; P03: “*I would not want the Wi-Fi to be missing, current, some things may not be correct”*; P06: *“I would be worried in case of a blackout of the entire system”*). In part, this problem has been addressed by one of the participants with a possible solution. She mentioned the presence of manual controls (i.e., walls buttons) that will allow controlling the smart home also if a Wi-Fi connection was not present (P04: “*The not working is a risk, but having the manual part is reassuring*”). These malfunctions, however, are considered more serious when they involve systems for personal safety. Two operators underlined in such circumstances potential severe but unreported risks (P01: “*a sensor may not work, this is also a potential risk”*; P03: *“some emergencies, I do not know for example a fire by magnifying, they are not declared in the app exactly”*). One operator reported the need to be able to call support after trying unsuccessfully to solve a problem by herself (P03: “*first, I try to understand what is not working. I evaluate the situation when the app does not work, and if I find myself in a difficult situation that I cannot solve alone, then clearly yes, I have to call someone, but it concerns events that I hope are important and not in small things*”). The interviews also pointed out the risks from external attacks (P03: *“I think that afterward, it will be up to the technicians to study a security element to avoid external infiltration into applications concerning everything they have to guarantee”*). It should be noted that only one participant reported it, showing a generally low awareness of cybersecurity problems.

*Q3 - According to you, is it simple to become quickly proficient in using the application and the home automation system?*

Overall, the participants reported that the control interface was easy to use (P02: “p*racticing yes”*; P03: “*In my opinion yes*”) and intuitive (P01: “*there are intuitive elements*”; P02: “*the system is intuitive, the system is intuitive*”; P04: “*Even scenarios are intuitive*”) and that it is possible to learn how to use the application with a short period of practice (P01: “*with a bit of training you can do it*”; P02: “*when you use it, it becomes more automatic*”; P04: “*continuing to use it becomes easier and easier*”; P07: “*with a bit of training it became a natural interaction*”; P06: “*Yes, after you use it a couple of times*”). One participant (P05) was more enthusiastic. She stated that the app and IoT technologies were simple (“*I liked it, it’s simple*”), fun (“*It was also fun, I must say*”), quick to learn (“*you learn it quickly*”), and highly usable (“*It’s clear. Is explained clearly and it’s easy to use*”). The influence of age and technology expertise emerged in the answers (P02: “*those who are younger are already born with the instrument and have a different history and are certainly more skilled*”; P03: “*they are used to the smartphone… they will be able to use it even better than me*”). Besides, for the first time, the importance of personal technological predisposition was mentioned (P04: “*I believe that there is always the most and the least capable persons*”).

*Q4 - Do you think that it could be possible to leave some of your working responsibilities to the home automation system?*

In general, despite the answers indicated a positive attitude to delegate working responsibilities, caregivers affirmed that they would leave the system with the most practical and low-responsibility tasks (P01: *“In part yes, it can be in control of some situations, of some tasks yes, it is very practice”*; P03: *“More than responsibility I would say for some tasks*”; P05: *“Watching television; open the windows if they need to, get food”*; P06: *“Yes to those more futile things yes. That is in the sense of turning on the light, doors, these things here.”*; P07:*” He can safely turn on the lights or check the gas, air, or anything else. I think he could easily handle work duties as well”*). The main reason is that they felt the responsibility of actively supporting and grant the safety of people with disabilities (P03: *“Not for the work that I do, I deal with people, not with objects or materials, I do not want to give all the responsibility to a home automation device I tell you the truth “*; P06: *“Not when is linked to the person safety”*). The concept of not leaving all their work duties to the system can be explicitly found in the majority of the sample (P01: “*However, if I think about security surveillance and other aspects, I still need time to rely on the system fully, I should have something”*; P03: *“Partially yes, absolutely, but the responsibility in the first place must be mine”*; P04: *“the complete 100% no”*). In one comment, this concept can be inferred (P02: “*Then surely the application gives the possibility of being less present as surveillance”*). Her statement does not take surveillance/assistance for granted but indicates how a smart integrated system gives the opportunity of being less present. One caregiver suggests using IoT systems video cameras to surveil residents when they have to leave the apartment temporarily (e.g., going quickly to the grocery store, P01: “*video control could give greater security*”). Other two caregivers stated that they would be more prone to trust a system that allows people with disabilities to call for help in case of need (P01: “*knowing that one of the people can effectively call or activate independently*”; P04: “*at least I’m sure that a child with this device here can give the alarm or thanks to it call me with the tablet or the like for the emergency*”). This question also points out insights about the Q1. Indeed, two of the operators underlined the system usefulness to reduce work-related stress thanks to the active surveillance and possibility for people with disabilities to ask for help through the IoT system (P01: “*I would leave people with disabilities in here [in the apartment], and I can go away, I can go and get something*”; P02: “*Then surely the application gives the possibility to be less present as surveillance*”; P04: “*that time when I have to go out for a moment I go away more calmly*”).

## Discussion

This work described a preliminary trial in the context of the Domho project, involving a sample of seven caregivers in using a mobile application that permits the control of different smart devices of an integrated IoT system installed inside a residential apartment. Participants carried out four tasks designed to examine the performance, user experience, and usability of a control interface designed and developed in DOMHO. Besides, the subjective perceptions of caregivers towards Smart Home and IoT systems were assessed.

**Figure 8:**
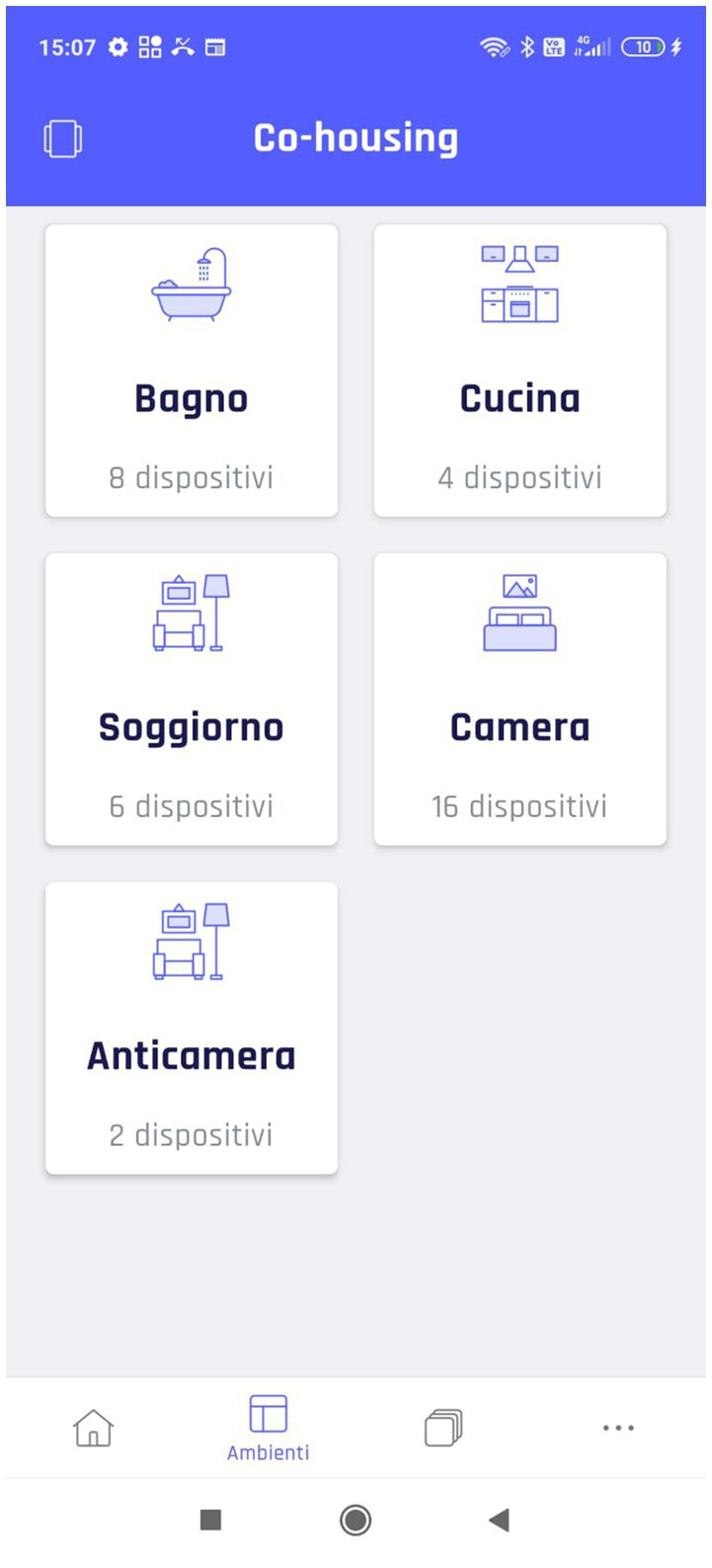
Subdivision of the SH environment.

Regarding video analysis, the first result that emerged is the importance of the organization of the living spaces. In T1, 5 out of 7 participants tried to manage lights and automation by selecting the kitchen instead of the living room. This occurrence is linked to the fact that the user interface splits the day area into two parts, i.e., kitchen and living room (Figure 8).

This result showed that this configuration causes confusion and slows down the interaction with the smart devices insofar as caregivers considered the wide room as a single open space. Thus, they select the wrong “sub-room” in trying to activate lights or automation. Instead, the control interface organization should be intuitive and clear without requiring users to remember information (Sharp et al., 2019). Using two labels to describe different sub-spaces inside the same room (i.e., open space), even if the system uses known conventions, might negatively influence the interaction. Indeed, participants must remember the exact technologies present in each part of the open space.

Another aspect that emerged from the video analysis of T3 (i.e., bedroom manual control) is the importance of allowing end-users to customize the labels inside a control interface. T3 presented the longer time on task (*M*=241s; Table 2) likely because the smart devices labels were selected by the developers and not directly by the operators. T3 breakdowns were caused by difficulties in comprehending the different labels assigned to the smart lights of the beds. Nevertheless, the DOMHO application allows the possibility to customize the names of devices and living spaces (i.e., kitchen, living room) according to the user’s preferences. This aspect is even more relevant whether individuals with disabilities are considered. In this case, personalization in terms of simplification is crucial to increase the control interface accessibility and inclusiveness (Loitsch et al., 2017; Estes et al., 2020).

**Figure 9:**
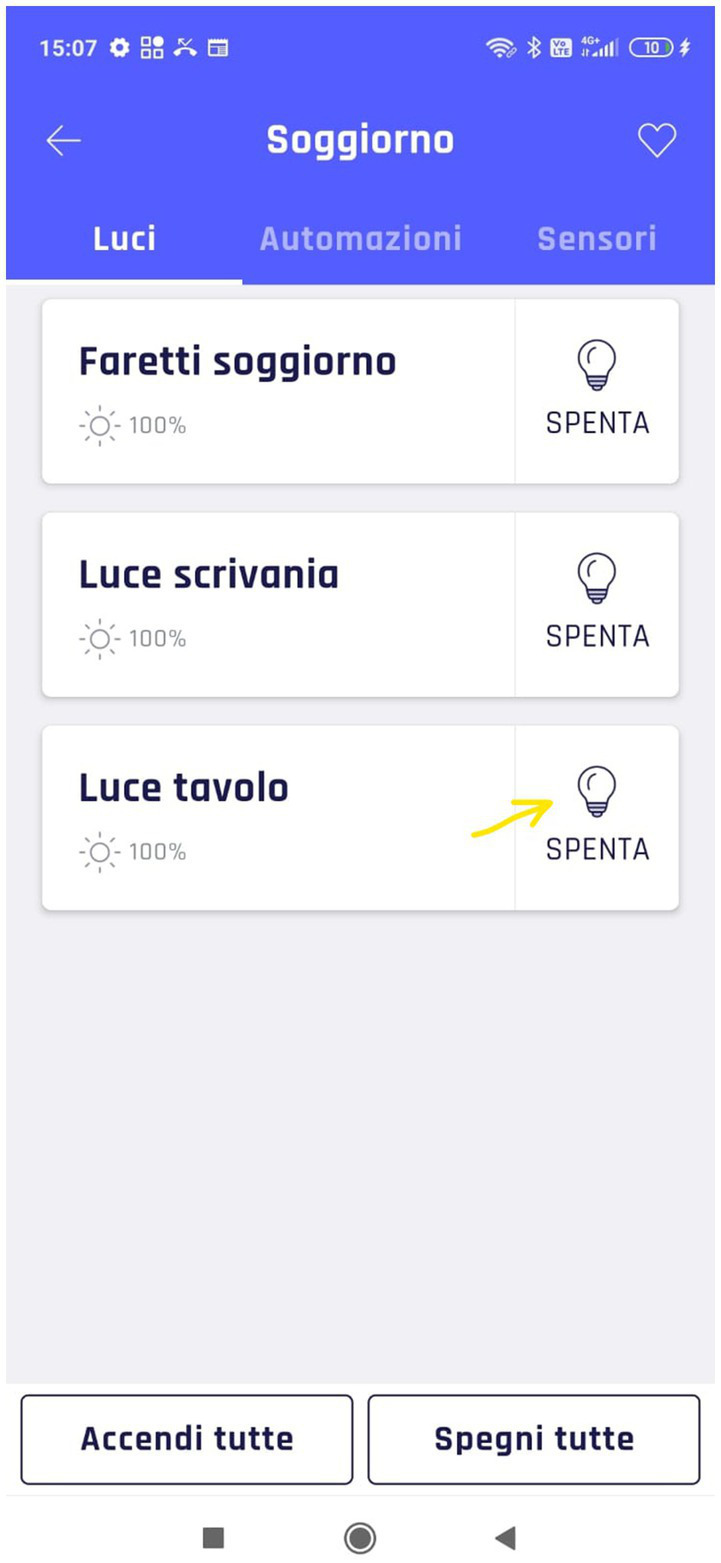
Shortcut for switching on lights.

Another aspect of usability that should be present in these types of applications is the flexibility of use. According to the ten Nielsen Heuristics, the interaction should be flexible and efficient, easy to use for the novices, and present alternative ways to accomplish the same action and shortcuts for expert users (Nielsen, 2005). The video analysis shows that during the turning on of the living room lights, one participant (P04) did not click on the white part of the button (like the other participants) but found a shortcut clicking on the lamp icon (placed on the right part of the button) to turn it on instantly (Figure 9), reducing the number of taps. However, this result shows that the application is designed to allow the accomplishment of the same task in alternative ways exploiting intuitive icons that might speed up the interaction based on the user expertise (i.e., novices, experts; Sharp et al., 2019).

**Figure 10:**
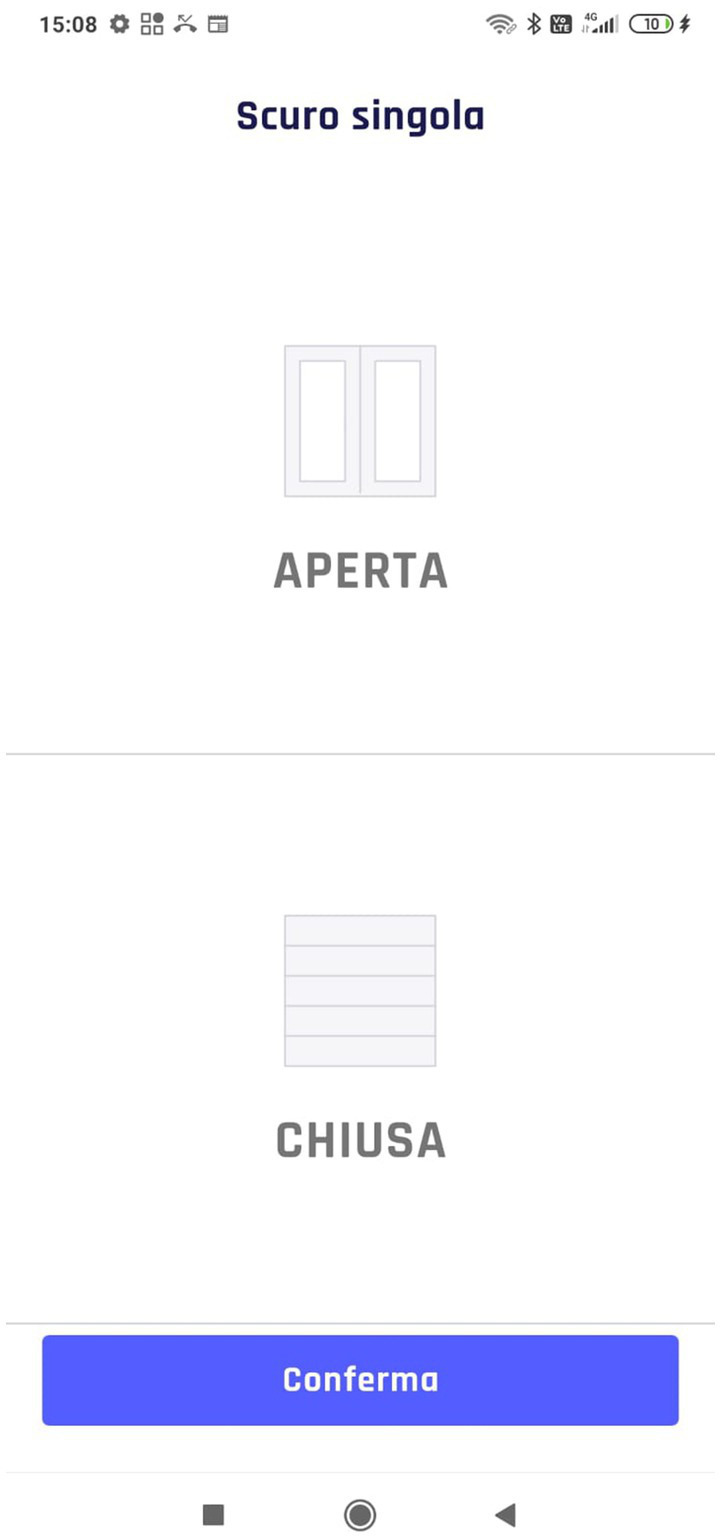
Automation feedbacks.

**Figure 11:**
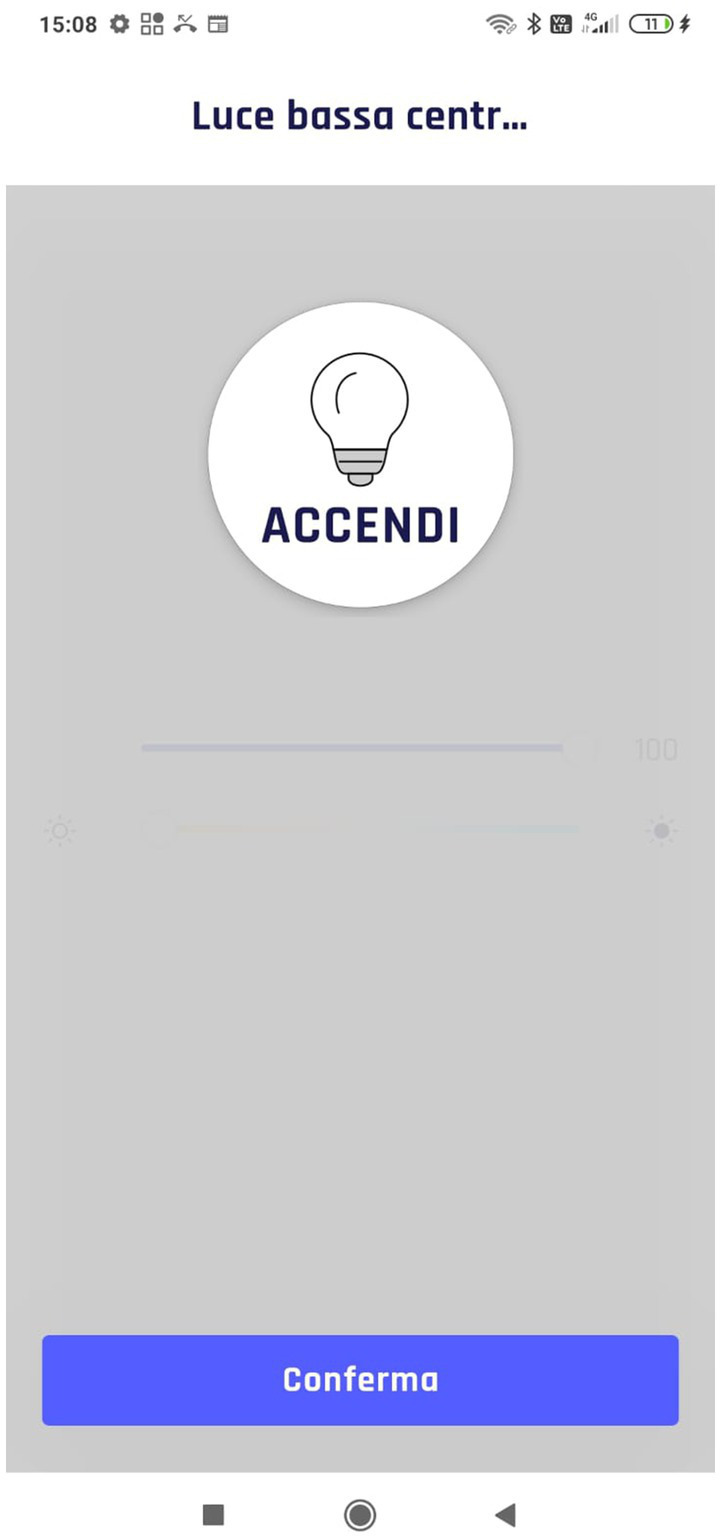
Light off feedback.

**Figure 12:**
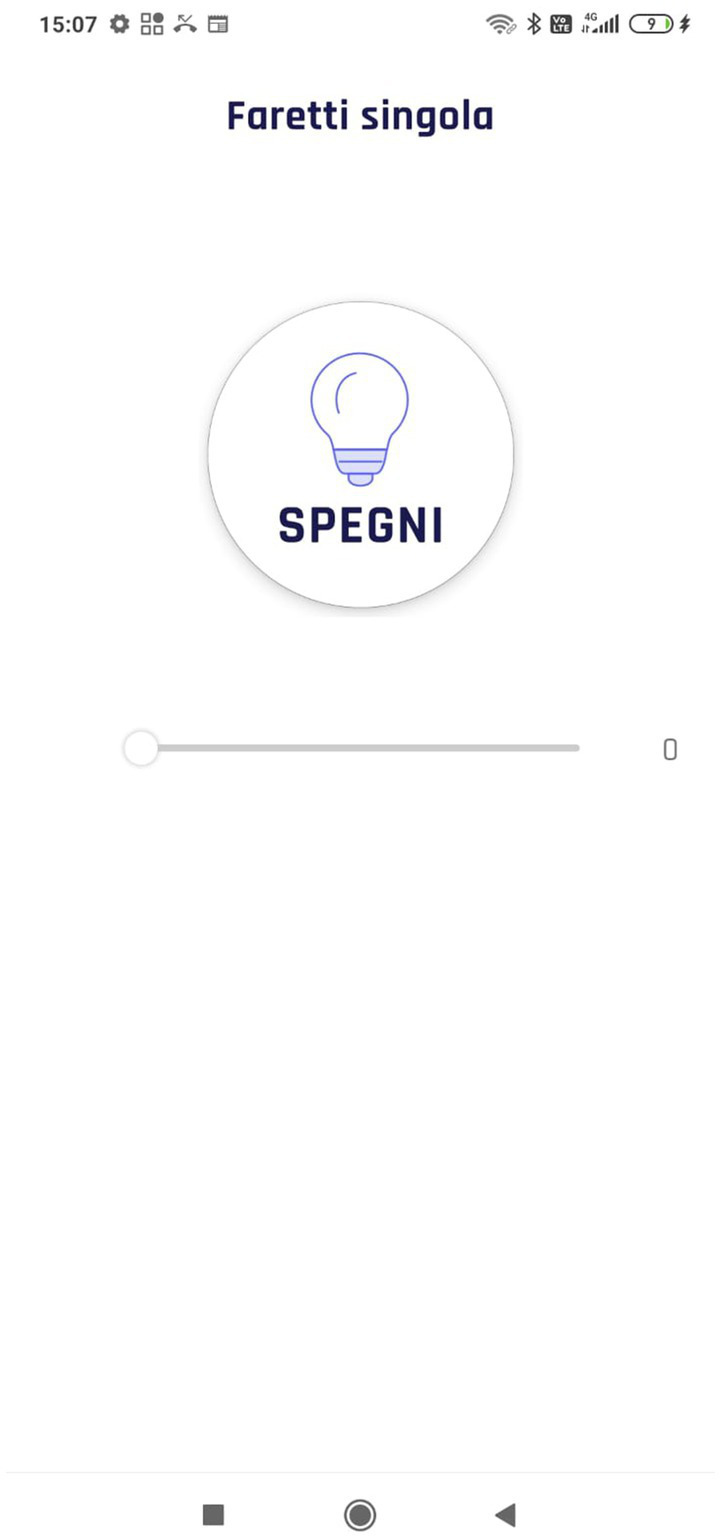
Light on feedback.

One of the main problems encountered by caregivers was setting up a manual or automated scenario without controlling the settled state with an appropriate feedback. For this reason, two breakdowns occurred. In T2, 3 out of 7 participants turn off lights instead of turning them on, failing to accomplish a part of the task. This lack of feedback and interaction-related problems are underlined by the lower percentage success in T2 and T4 (Figure 4, T2=67%, T4=89%). In particular, the analysis shows the difficulties in understanding the current lights state. However, it was not the same for automation. As can be noticed from the comparison in Figures 10, 11, and 12, the difference was precisely in the type of feedback. For the automation, the screen presents the user with the possible states (Figure 10). However, in lighting, the system uses a method more based on logic and text. If the light is set off, the system offers the user a screen with a dark background and a message “turn on” (Figure 11). Instead, when it is set as on, it presents a light background and the words “turn off” (Figure 12).

Despite being a system that follows a precise logic, the interface is confusing for a novice user, as demonstrated by the analysis. Therefore, it is advisable always to show the user immediate, clear, and understandable feedback, based mainly on the graphic component and not on the logic language rules. Indeed, clear icons accompanied with text are particularly indicated for novice users to reduce the mental load needed to learn the new technology, especially when these people, such as the elderly, have some impairments (Huang et al., 2019). Although the experiment was conducted only with the operators, it is also helpful to extend this consideration to the other type of end-users who will use the system, namely individuals with disabilities. Given the problems of understanding due to potential mild cognitive disabilities, these people could also benefit from using graphical elements (i.e., icons).

An overall positive subjective experience emerged from the analysis of the UX questionnaire. Interestingly, the median scores assigned to privacy, trust, and security dimensions, that represent well-known issues in the IoT field (Atlam and Wills, 2020), were all above the median of the scale (i.e., Pri=5; Tru=4.5; Sec=3.5). A possible explanation could be that because the operators were involved in developing and selecting the devices (i.e., participatory design approach) included in the smart co-housing apartment. Together with the sense of usefulness perceived about the system, their involvement during the design phase could have resulted in an overall positive attitude towards the DOHMO application and IoT system. The interviews data also support this. Also, the multiple clarifications regarding the policies of personal data protection guaranteed by the researchers and companies involved in the project increased the caregivers’ confidence in the system ability to protect their data and privacy. Another possible explanation could be that the operators were not fully aware of the IoT system’s privacy problems. Summarizing, it seems that involving users actively in the design and selection of technologies has resulted in higher smart home trustworthiness. The interviews show that they were more prone to think about malfunctions and infrastructure problems when the researchers ask about system possible problems and limitations.

Finally, the questionnaire scores show a high level of pleasantness and satisfaction in using the system and highlight the intuitiveness of the system. These aspects could be related to the crucial involvement of participants in the design and development of the DOMHO integrated system. We look forward to assess these attitudes and subjective perceptions considering people with disabilities. Indeed, the scientific literature has underlined the importance of capitalizing on user centered design to co-create intelligent tools and environments with and without disabilities (Augusto et al., 2018; Chin et al., 2019). The analyses described so far regarding the user experience questionnaire and the behavioural data of tasks success percentage show that the participants evaluated the interaction positively, obtained satisfactory results, and evaluated it as usable, reliable, and able to guarantee security and privacy, as hypothesized in H1.

As for the analysis of the interviews, the results can be summarized as follows. In the answers to the first question, caregivers showed positive attitudes towards the system adoption as a supportive tool in their work. The reported advantages are reducing workload and enhancing the autonomy and independence of individuals with disabilities (Carnemolla, 2018). These comments align with the literature on caregivers and decrement in burden due to the exploitation of smart technologies in their working environments (Seelye et al., 2012). The analysis of the interviews’ transcriptions highlights how this perceived usefulness seems to be relevant for the envisioned benefits for both caregivers and the individuals that they assist.

Concerning the second question, main concerns emerged about generic system malfunctions and minor errors (e.g., lack of electricity, no internet connection, not working lights, etc.) that become more worrying when they regard the safety systems and, therefore, sensors (e.g., air quality, video cameras, etc.). This problem could be partially mitigated by providing alternatives to control the intelligent technologies, like manual control systems (i.e., wall buttons) and the possibility to control them without an internet connection.

As for ease of use, operators stated that the system and the interface are simple to learn and intuitive, even if they require a short period of practice to be mastered, reflecting a high level of learnability (Grossman et al., 2009). To be more specific, this is known as “initial learnability” which allows users to reach a reasonable level of efficacy and efficiency in utilizing a novel technology in a reduce amount of time (Nielsen, 2005). These findings matched the high scores assigned to the usability in the UX Questionnaire (Figure 7, Us=4). Among the factors that influence rapid learning highlighted by the interviews are age, expertise with technologies, and personal predisposition. The video analysis results also confirmed this impression of the operators, confirming that the number of errors made is influenced by the participants’ age (Figure 6).

Finally, as far as professional responsibility is concerned, the system seems to have been well accepted but cannot completely fulfil the operators’ responsibilities. To better define this concept, it emerged that the system is particularly suitable for manual, simple, and repetitive tasks. Nevertheless, it does not generate blind trust in the operator in case of possible risk situations for people’s health. Despite this limit, there was a positive attitude towards the intention of adopting this integrated smart system in the future to prevent dangerous situations. Nevertheless, the system is perceived as a “technological collaborator” that has to be supervised in the most important, complex, and delicate tasks. As for the possible solutions to enhance trust in the system during emergency management, the operators suggest that the system should be structured in such a way to ensure high accessibility for people with disabilities to call for help and receive quick assistance. The operators assign great importance to this concept of leaving the apartment in case of need. This behaviour could only be possible if at least one of the people with disabilities could set off an alarm. Therefore, putting the system in the position of empowering one of the occupants with disabilities to call for help could reduce the caregivers’ work-related stress and anxiety (Bruno et al., 2018). As for the video surveillance solution, the potential problems probably outweigh the benefits. Indeed, the security and privacy issues and the feeling of being controlled, that may be experienced by people with disabilities, could compromise the whole system’s acceptance and decrease the feeling of independence (Krempel and Beyerer, 2014).

Concluding, the interviews showed that the system is perceived as a positive instrument by the operators, who found it reliable, easy to learn and use. Furthermore, the perceived risks were minimal and mostly related to the infrastructures and not to the system itself. These results corroborate the H2 and therefore show the maturity of these systems for introduction into real work environments.

## Conclusion

This study firstly highlights some of the characteristics that similar systems should present to elicit a positive user experience and be accepted by caregivers, such as flexibility in the terminology and organization of a control interface elements, the presence of appropriate feedbacks and so on.

The research also highlights that the whole assisted living environment has been well accepted by the caregivers. Moreover, the study hypothesizes that even known problems in the field of IoT technologies, such as trust and privacy, can be mitigated by involving the participants in activities of participatory design. Besides, moderator factors in the acceptance of these advanced technologies are the perceived utility and usefulness in work supporting and in increasing life quality and well-being of the assisted persons. Future trials will involve individuals with disabilities to assess user experience, usability, acceptance of this smart co-housing apartment. Groups of two/three individuals on rotation will live for 2/3days (i.e., weekends) inside this Smart Home with one caregiver. Specific attention will be devoted to the subjective perceptions of living in a smart environment, QoL, satisfaction, autonomy and independence, and the co-housing experience itself. Despite the major limitation of this study, namely the participants numerosity, using a set of mixed research methodologies (i.e., quantitative and qualitative) allow a comprehensive analysis of the overall caregivers’ experience and performance in interacting with a smart home and its control interface. Designers and developers could benefit from these indications to realize technologies that meet the users’ needs, both for people with disabilities and their caregivers.

## Data Availability Statement

The original contributions presented in the study are included in the article/Supplementary Material, further inquiries can be directed to the corresponding author/s.

## Ethics Statement

The studies involving human participants were reviewed and approved by Human Inspired Technology Research Centre Ethical Comitee. The patients/participants provided their written informed consent to participate in this study.

## Author Contributions

DB, PP, and AZG contributed to the study design and data collection and analysis. DM, AS, AZ, and LG wrote sections of the article and contributed to the design of the study. All authors contributed to manuscript revision, read, and approved the submitted version.

## Conflict of Interest

The authors declare that the research was conducted in the absence of any commercial or financial relationships that could be construed as a potential conflict of interest.

## Publisher’s Note

All claims expressed in this article are solely those of the authors and do not necessarily represent those of their affiliated organizations, or those of the publisher, the editors and the reviewers. Any product that may be evaluated in this article, or claim that may be made by its manufacturer, is not guaranteed or endorsed by the publisher.

## Supplementary Material

Supplementary Table 1 UX Ad Hoc questionnaire and investigated dimensions.
