# Supplementary material for: Smart Co-housing for People With Disabilities: A Preliminary Assessment of Caregivers’ Interaction With the DOMHO System
Source: Front Psychol. 2021 Sep 3;12:734180. doi: 10.3389/fpsyg.2021.734180 (PMC8446196; doi:10.3389/fpsyg.2021.734180)
Supplement: Supplementary Table 1 — UX Ad Hoc questionnaire and investigated dimensions. [file Table_1.DOCX]

UX AD HOC QUESTIONNAIRE

1. I believe that this application is easy to use. (USABILITY)
2. I think adding a new automatic scenario in the application is easy. (USABILITY)
3. I think that external parties cannot access the data I entered in the application. (PRIVACY)
4. I think asking for help with this application is easy. (USABILITY)
5. I believe that partially lowering the curtain with the application is easy. (USABILITY)
6. Important functions are clearly visible in this application. (VISIBILITY & SYSTEM STATUS)
7. I think finding the information you need is easy. (VISIBILITY & SYSTEM STATUS)
8. I believe that the application is reliable. (SECURITY)
9. Using this application was frustrating. (SATISFACTION - REVERSED)
10. Using this application was enjoyable. (PLEASANTNESS)
11. I felt confident in using the application. (USABILITY)
12. I think adding a new manual scenario in the application is easy. (USABILITY)
13. Overall, I am satisfied with how I used this application. (SATISFACTION)
14. I think the smart apartment collects too much information about me. (PRIVACY - REVERSED)
15. I think I could entrust some of my job responsibilities to the system that controls the smart apartment. (TRUST)
16. I would like to use this application again. (PLEASANTNESS)
17. I think that the functioning of the application is transparent. (TRUST)
18. The fonts are clearly legible. (VISIBILITY & SYSTEM STATUS)
19. Finding the information I need within the application is complicated. (VISIBILITY & SYSTEM STATUS - REVERSED)
20. The labels used by the application is easily understandable. (RECOGNITION RATHER THAN RECALL)
21. The icons used by the application are easily understandable. (RECOGNITION RATHER THAN RECALL)
22. I think adding a new event-triggered scenario in the application is easy. (USABILITY)
23. I believe that using the application is safe. (SECURITY)
